# Ionizing Radiation, Higher Plants, and Radioprotection: From Acute High Doses to Chronic Low Doses

**DOI:** 10.3389/fpls.2018.00847

**Published:** 2018-06-26

**Authors:** Nicol Caplin, Neil Willey

**Affiliations:** Centre for Research in Biosciences, University of the West of England, Bristol, Bristol, United Kingdom

**Keywords:** ionising radiation, radiobiology, environmental protection, DNA damage, oxidative stress, plant stress

## Abstract

Understanding the effects of ionizing radiation (IR) on plants is important for environmental protection, for agriculture and horticulture, and for space science but plants have significant biological differences to the animals from which much relevant knowledge is derived. The effects of IR on plants are understood best at acute high doses because there have been; (a) controlled experiments in the field using point sources, (b) field studies in the immediate aftermath of nuclear accidents, and (c) controlled laboratory experiments. A compilation of studies of the effects of IR on plants reveals that although there are numerous field studies of the effects of chronic low doses on plants, there are few controlled experiments that used chronic low doses. Using the Bradford-Hill criteria widely used in epidemiological studies we suggest that a new phase of chronic low-level radiation research on plants is desirable if its effects are to be properly elucidated. We emphasize the plant biological contexts that should direct such research. We review previously reported effects from the molecular to community level and, using a plant stress biology context, discuss a variety of acute high- and chronic low-dose data against Derived Consideration Reference Levels (DCRLs) used for environmental protection. We suggest that chronic low-level IR can sometimes have effects at the molecular and cytogenetic level at DCRL dose rates (and perhaps below) but that there are unlikely to be environmentally significant effects at higher levels of biological organization. We conclude that, although current data meets only some of the Bradford-Hill criteria, current DCRLs for plants are very likely to be appropriate at biological scales relevant to environmental protection (and for which they were intended) but that research designed with an appropriate biological context and with more of the Bradford-Hill criteria in mind would strengthen this assertion. We note that the effects of IR have been investigated on only a small proportion of plant species and that research with a wider range of species might improve not only the understanding of the biological effects of radiation but also that of the response of plants to environmental stress.

## Introduction

There has been much recent interest in the health of organisms at radioactively contaminated sites such as those at Chernobyl and Fukushima. Thriving communities of flora and fauna (e.g., Deryabina et al., 2015) have surprised many people but there are also reports of significant effects of chronic irradiation at surprisingly low doses (e.g., Boratyński et al., 2016). This ‘paradox’ persists, in part, because the observed effects on organisms from different doses of environmental radioactivity have yet to be synthesized into a coherent understanding. It is important to do this because ionizing radiation (IR), whilst occurring naturally, is a pollutant, both actual and potential, from a nuclear industry of global significance – at the beginning of 2018 there were numerous polluted nuclear-legacy sites and 448 on-grid civil nuclear reactors generating >10% of the world’s electricity, with 58 under construction (International Atomic Energy Agency [IAEA], 2018) and many more planned. Almost all of the highly active nuclear waste ever generated is yet to be stored in permanent repositories, and credible environmental safety cases will be necessary prior to their construction. If nuclear power is to be a significant source of low carbon electricity in the future and if we are to deal with the nuclear legacy and any further nuclear accidents or detonations, it is desirable to demonstrate that the effects of IR on flora and fauna are understood. This review is based on a compilation of current data for plants that, we suggest, helps to resolve the ‘paradox’ of the effects of IR in the environment by analyzing the available data within a stress-response context and then uses this to propose new contexts for research.

## Ionizing Radiation and the Evolution of Plants

Few biological phenomena can be fully understood without knowledge of their evolution. From an evolutionary perspective IR is a primordial stressor. Life on Earth evolved in varying natural background IR of cosmic and geologic origin. The activities of β and γ radiation from geological sources have decreased by about a factor of eight since the origin of life between 3.5 and 4 Ga ago (Karam and Leslie, 1999). Eukaryotic life (Archibald, 2015) probably began >2.5 Ga ago under conditions that received five times current background levels of β/γ radiation. When plants first colonized the land surface (*c.* 460 Ma ago) (Gensel, 2008) background IR levels were still significantly higher than at present. These figures are global averages – if background IR varied spatially as much in the past as it does now, many early life forms were exposed to much higher background IR than was average at the time. If life’s early exposure to IR helped drive the evolution of processes, such as DNA repair, that have found important roles ever since, it might help to explain the current occurrence of radio-resistance, and sometimes even the ability to adapt to radiation, in some extant prokaryotes (Siasou et al., 2017).

**FIGURE 1:**
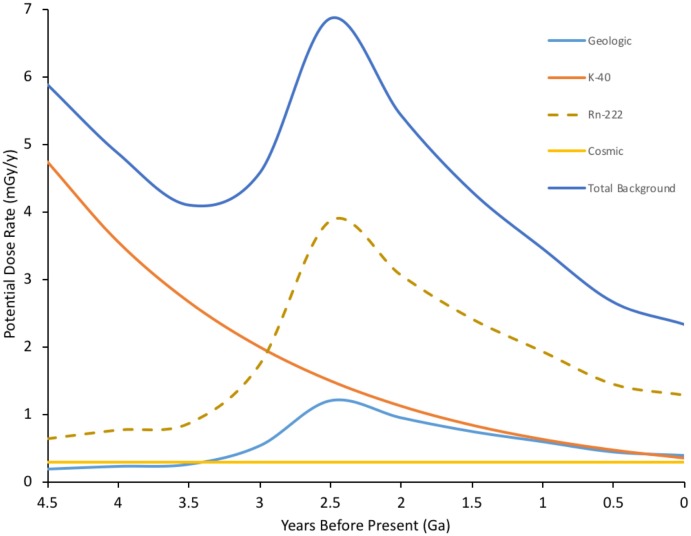
Estimated ionizing radiation (IR) dose rate through geological time at the Earth’s surface. Geological estimates based on β+γ doses taken from Karam and Leslie (1999). Rn-222 is a significant current contributor to background radiation doses (c. 1.2 mSv/y current global average) for inhabitants in contained environments on the Earth’s surface, including plants in canopies, which have evolved to exchange gases in the near surface environment. Rn-222 contribution to dose rate is, therefore, included and is estimated from geologic background, which is dominated by U decay series radioisotopes. K-40, estimated from its half-life, dominates internal doses to organisms and is thus a proxy for them. Total estimated dose rate is combined internal and external dose for an organism at the Earth’s surface – although, of course, for much of the last 4.5 Ga there were no organisms at the Earth’s surface. The geologically driven peak reflects events in the Earth’s crust including the formation of continental plates. Current global mean background dose rate is 2.5 mGy/y. (Details of calculations in **Supplementary Data Sheet S1**).

Estimating external doses of background radiation at the Earth’s surface depends on understanding geological events, with the coalescing of crustal plates probably the most important (**Figure 1**). About 460 Ma ago plants did not just colonize the land surface but also the above-surface atmosphere (Willey, 2016). They did this using morphology of increasing leaf area index, that not only increased light capture but also allowed increased exchange of gases with the near surface atmosphere. In addition to background β and γ, ^222^Rn contributes very significantly (>60%) to current background doses to humans (Health Physics Society [HPS], 2015) and we suggest, therefore, that during their evolution it may also have contributed to doses to some higher plants, especially those inhabiting canopies with low air flow. Further, we note how important to understanding the effects of IR on higher plants that the exposure of ancient prokaryotes to IR might be – the key to the success of higher plants is that they house plastids of prokaryotic origin (mitochondria and chloroplasts) at the Earth’s surface-atmosphere interface. It is, therefore, estimated that average background doses in the range up to 7 mGy/y (c. 20 μGy d^-1^) occurred for a significant period of the evolution of plant life but that high background areas may have had significantly higher dose rates.

When energy from IR is deposited directly into DNA it can damage it. There are numerous chemical and physical processes that can damage DNA in a variety of ways, but IR is one of the few that can induce a range of damage, including double stranded breaks (DSBs) (Oladosu et al., 2016). Factors that cause damage on a single strand probably helped favor the evolution of a double-stranded molecule as genetic material – a second strand provides a template for repair of damaged bases or nucleotides (Freidberg, 1997). Multiple copies of chromosomes underpin further processes of DNA repair – for example homologous recombination (HR), which in many eukaryotes helps produce variation in haploid gamete cells during meiosis, is also involved in the repair of DSBs (Jackson and Bartek, 2009). Homologous pairing, and hence an important DSB repair pathway, is promoted in archaea by *RadA*, in bacteria by *RecA* and in eukaryotes by *Rad51*, which are slightly different versions of the same gene in all organisms – eukaryotic nuclear DNA probably acquired *Rad51* via transfer of *RecA* from prokaryotic endosymbionts (Lin et al., 2006). *Rad51* was identified through its radiation responsiveness although IR was not necessarily the DSB-causing agent that drove its evolution. It has long been suggested that there is a link between DNA damage and the evolution of sex (Bernstein et al., 1985; Rocha, 2016) with *RecA* having a crucial role in both (Bernstein and Bernstein, 2010). Overall, the ubiquitous, and esthetically appealing, static image of the double helix of DNA detracts from the reality of dynamic processes of DNA damage and repair that underpin life on Earth (Friedberg, 2003) and that evolved in response to primordial stressors, perhaps including IR. Direct effects of background IR on DNA are probably less significant now than they have ever been but, especially in ancient high background areas, they may have played a role in the evolution of both the genetic architecture and the DNA curation processes of life.

**FIGURE 2:**
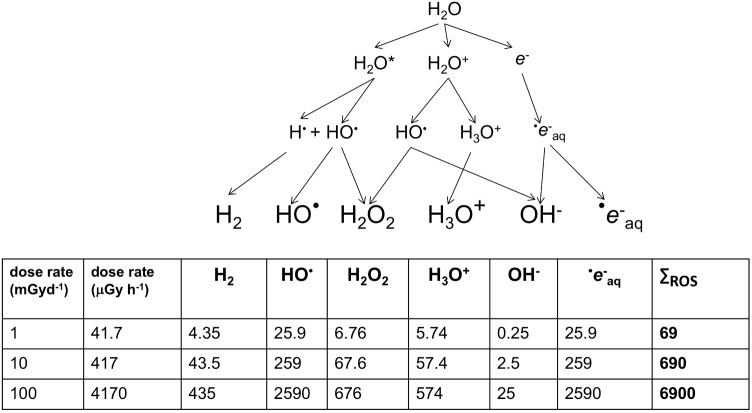
The products of the radiolysis of water (×10^-16^ mol/g). Smith et al. (2012) describe the above cascade for the production of oxidizing species by radiolysis. During chronic irradiation, several of the molecules produced react continuously to give the products shown above. For each product, *G*-values describe the relationship between energy deposited and the amount of product produced. *G*-values for β/γ radiation from Cs-137 were used to calculate, in ×10^-16^ mol/g, the amount of product at a range of dose rates. HO is short-lived but strongly oxidizing and e^-^_aq_ (a solvated electron) can combine with O_2_ to produce dioxygen radicals (O_2_^-^ – ‘superoxide’). The consequences of HO and e^-^_aq_ production dominate the oxidative effects of radiolysis on organisms.

**FIGURE 3:**
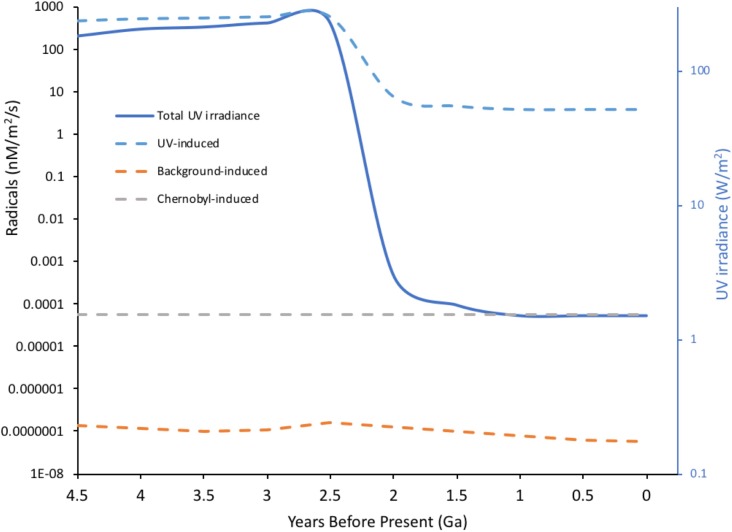
Radical induction-potential from water by different radiation sources through Earth’s history. UV radiation with λ > 100 nm is not energetic enough to directly ionize water but π-bonds and *n*-electrons in organic molecules can absorb UV, producing exited molecules that, in aqueous solution, can induce the formation of radicals from water. Radiation-induced chemical yields from ionization (*G*-values in moles per 100 eV energy deposited) were used to calculate potential radical production from both UV and background radiation through Earth’s history. For UV acting on organic molecules *G* = 0.01 was, conservatively, assumed and for background radiation acting on water *G* = 2.8 (the value for Cs-137 emissions). For UV, current energy in the 250–350 nm range was taken as 1.5 W/m^2^, converted to eV and an estimate of variation in total geological irradiance of UV (Cockell and Horneck, 2001) used to calculate the potential for radical production. For comparison, the potential for Chernobyl radiation to induce radicals was calculated assuming 1MBq of Cs-137/m^2^ – an activity that occurs widely in the Chernobyl Exclusion Zone. For Cs-137 an energy of 1.127 MeV per Bq was used to include both β and γ emissions. The massive drop in potential radical production from UV at Earth’s surface reflects the formation of the ozone layer. The concentrations of radicals to which life was actually exposed is not necessarily directly related to the predictions above because: constituents of the Earth’s atmosphere other than ozone, which have changed significantly over time, can affect UV penetration to the surface; early organisms may have lived in significant depths of water; life probably evolved UV screening molecules at an early stage. (Details of calculations given in **Supplementary Data Sheet S2**).

Ionizing radiation can also damage DNA indirectly via the products of radiolysis, which causes a cascade of reactive molecules (**Figure 2**). Many of these molecules play key roles in the processes of life, their reactivity making them useful in signaling and defense but also potentially damaging to biomolecules (Foyer and Noctor, 2016). The reactive oxygen species (ROS) resulting from radiolysis of water are important in producing its effects at high doses, including for example during radiotherapy or corrosion of pipes in nuclear reactors. In an aqueous environment, e.g., cells, ROS production can be calculated from dose rates (Smith et al., 2012; **Figure 2**). However, during the evolution of life, UV, which can also cause direct DNA damage, has been a much more significant source of ROS than IR. UV-C with a wavelength below 100 nm is ionizing but is also absorbed by many atmospheric constituents, perhaps including some that occurred in the early atmosphere (Hessen, 2008), and has likely never been a particularly significant source of ROS in aqueous environments, including cells, at the Earth’s surface. UV with wavelengths longer than 100 nm does not generally ionize water but can ionize other organic molecules, including proteins. In an aqueous solution, these photoionized molecules can induce the production of ROS from H_2_O (Pattison and Davies, 2006). The probability of this occurring is relatively low compared to the probability of radiolysis induced by IR but the amount of UV arriving at the Earth’s surface is, even after the formation of the ozone layer, much more significant than the amount of background IR. Calculations of the production of ROS produced by UV over geological time compared to that from IR suggest that UV has, throughout evolution, been the most significant radiative source of ROS that organisms have had to contend with (**Figure 3**). Overall, understanding the effects of IR must occur with recognition that it was a feature of the primordial environment that is now less intense than it once was and that there are other radiative stressors that can damage DNA and promote the formation of ROS, often at much more significant rates, than does IR.

## Environmental Protection and the Dose-Effects Data

In general, data from accidents and controlled experiments suggest that, with some differences between species, acute high doses of IR in the range of 10–1000 Gy can be fatal to plants (United Nations Scientific Committee on the Effects of Atomic Radiation [UNSCEAR], 1996). Although fewer studies have examined chronic low dose effects of IR in plants, United Nations Scientific Committee on the Effects of Atomic Radiation [UNSCEAR] (1996) suggested 10 mGy/d (417 μGy h^-1^) as a threshold dose rate for radio-protection of plants (Nelson-Beyer and Meador, 2011). This confirmed a long-established International Atomic Energy Agency (IAEA) threshold for radiation dose rates of <10 mGy d^-1^ having ‘no detrimental effects’ for populations of terrestrial plants in the field (International Atomic Energy Agency [IAEA], 1992). To help account for differences in response between different organisms, including different types of plant, the International Commission on Radiological Protection developed (International Commission on Radiological Protection [ICRP], 2008) the use of a set of reference animals and plants (RAPs). These were later supplemented with DCRLs for each RAP (International Commission on Radiological Protection [ICRP], 2014) – a range of dose rates that might prompt evaluation of potential radiological impacts. The ICRP’s RAPs include plant DCRLs for grass of 1–10 mGy d^-1^ (41.7–417 μGy h^-1^) and for pine trees of 0.1–1 mGy d^-1^ (4.17–41.7 μGy h^-1^). The grass RAP provides a reference range for herbaceous higher plants and pine trees a reference range for the more IR-sensitive woody plants. The EU-funded ERICA project suggested, after including a safety factor of 5, a chronic exposure screening value of 10 μGy h^-1^ for ecosystems (Garnier-Laplace and Gilbin, 2006). Ecosystems will, however, include some organisms that are more sensitive than plants. Here we focus on discussing published data on the effects IR on plants with an ultimate focus on the DCRLs for grass and pine RAPs because they are a well-developed international framework for protecting plants from the effects of IR.

There are several reasons for probing the appropriateness of these DCRLs. The development of RAPs emphasized that the understanding of the effects of radiation on plants is much less than that for humans or other animals. This continues to be the case and can, in part, be attributed to the challenges of studying radiological impacts on plants. For example, when studying pine trees, it can be challenging to establish either accurate external doses at different heights or accurate internal doses arising from accumulation in different parts of a large organism (International Commission on Radiological Protection [ICRP], 2008). Additional complications when studying plants, and about which relatively little is known, include the radio-sensitivity of different above- and below-ground organs (for example buds, roots, and root hairs), significant differences in life-span of different species and seasonality in responses. Further, in radiobiology, IR-induced effects are generally divided into deterministic effects that occur when a dose-threshold is exceeded and can be estimated by endpoints such as mortality, morbidity or reproductive success, and stochastic effects that are probabilistic and measured by endpoints whose incidence increases proportionately with dose (United Nations Scientific Committee on the Effects of Atomic Radiation [UNSCEAR], 2006). The importance of stochastic effects in plant radiological protection, especially at chronic low doses, is unclear. In 2005, a European Commission report suggested that, despite observed impacts on some individuals, stochastic effects arising from chronic low doses of IR may be of little relevance to protecting populations of non-human biota, although the report did acknowledge that effects at a population level are not well known (Björk and Gilek, 2005). This is in part because stochastic effects can produce differences between not only individuals but also, for example, between different parts of a plant (Esnault et al., 2010). This presents some statistical challenges not least because in plants with a small biomass data is often pooled from several individuals and many responses can be hidden. Esnault et al. (2010) suggested that there is a need for experiments to generate high definition intra-plant data. Such data are not yet available and the importance of stochastic effects to the protection of flora, although unlikely to be significant, are not clear.

**FIGURE 4:**
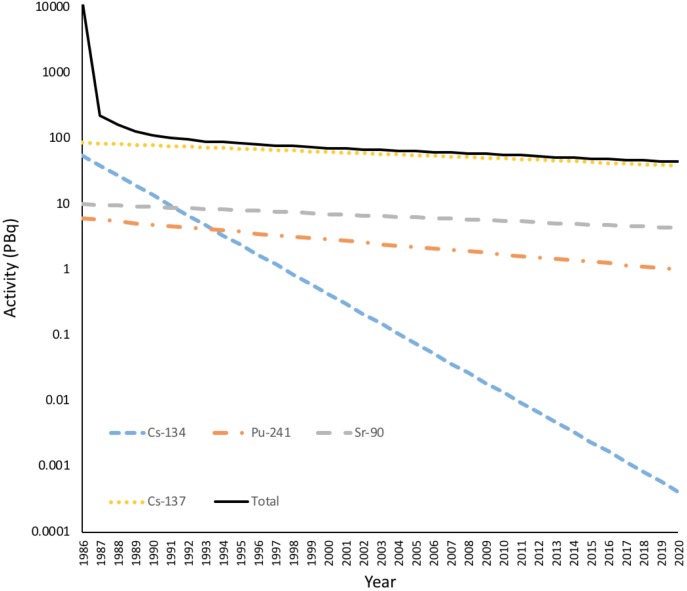
The Activity of radionuclides in the environment from the accident at Chernobyl. The total activity of radionuclides released from Chernobyl over a few days in 1986 was in excess of 11 EBq. Much of this was short-lived radionuclides such as ^33^Xe (6.5 EBq, λ = 5.3 days), ^132^Te (1.15 EBq, λ = 3.25 days), ^131^I (1.76 EBq, λ = 8 days), ^99^Mo (0.2 EBq, λ= 2.79 days), ^141^Ce (0.2 EBq, λ= 33 days). After a few years the remaining radioactivity was dominated by ^137^Cs, ^134^Cs plus some ^90^Sr and ^241^Pu. Radioactivity is now dominated by ^137^Cs. Many of the short-lived radionuclides are gaseous and emitted to the atmosphere but there was still a dramatic decrease in the dose to terrestrial organisms in the first year after the accident. (Full calculations given in **Supplementary Data Sheet S3**).

Further, many areas on Earth have a naturally enhanced background of IR (Saghirzadeh et al., 2008) and, for example, it has been suggested that the chronic exposure at Ramsar in Iran can have effects on plants up to a dose rate (4 μGy h^-1^) that is only about 10 times higher than the global average background (Ghiassi-Nejad et al., 2003) and is at the low end of the range of the DCRL for sensitive plants. Effects at similarly ‘ultra-low’ dose rates have been reported at Fukushima (Hayashi et al., 2015). In addition, many studies that have contributed to the development of DCRLs have used field locations with dose rate gradients as the basis for their research design. An association between existing environmental contamination and effects is only one indicator of cause, because locations with different dose rates can vary in other ways, often to an unknown extent, in both systematic and specific respects, i.e., there can be significant confounding factors. For example, due to the short-half lives of most of the radioisotopes emitted from the Chernobyl NPP (**Figure 4**) most contaminated locations with elevated dose rates post-1987 had much higher, and short-lived, dose rates during 1986 in the immediate aftermath of the accident. At Chernobyl, when attempting to assess the effects of a particular dose rate it can be difficult to separate any lasting effects of 1986–1987 dose rates from any effects of the post-1987 dose rates. Clearly, although there are established transgenerational effects of IR, in studies conducted a significant time after the accident this may be less of a complication.

**FIGURE 5:**
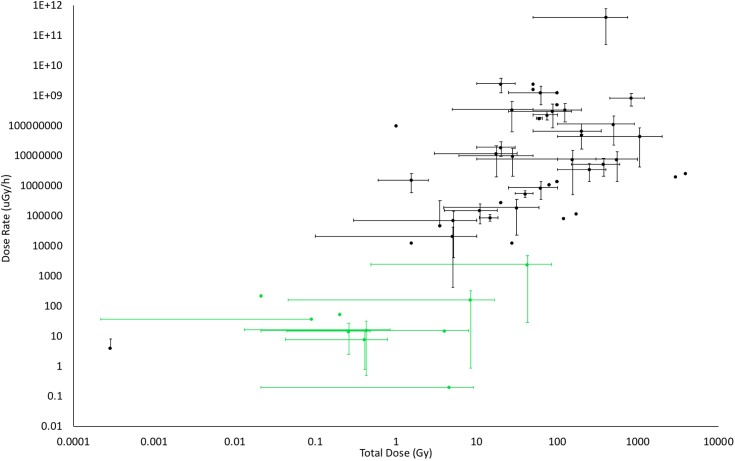
The doses and dose rates used in studies of the effects of IR on plants. Where possible, dose rates and total doses from published studies were determined from methods sections or by calculation of them from details provided. The bars on the points above represent the ranges of dose and/or dose rate used in the published works. Studies in the field are coded in green, those from the laboratory in black. Although not all published studies could be included because doses or dose rates were not provided or could not be calculated, this significant selection of the published data shows that there are few laboratory studies at low doses, especially for chronic exposures (Details of studies are in **Supplementary Data Sheet S4**).

In order to aid discussions of the effects of IR on plants, we compiled published studies of the effects of IR on plants and classified them according to exposure to IR (**Figure 5**). It is clear that there is a paucity of data on the effects of chronic low doses of IR on plants that were generated under controlled conditions. The studies that have investigated the effects of IR at contaminated sites clearly, and crucially for managing them, reveal what is happening at these sites under field conditions but they provide primarily associative evidence that the cause of any effects is exposure to chronic low-level IR. Published field studies of the effects of IR on plants are essentially epidemiological and, we suggest, attribution of cause should therefore meet the relevant criteria of causality. In epidemiology, the nine Bradford Hill criteria for establishing if association might be cause have not only a long-established use as…‘the most frequently cited framework for causal inference in epidemiology’… but also interpretations fit for the molecular age (Fedak et al., 2015). They are used in studies of the effects of IR on humans (McLean et al., 2017) and we suggest that they could be more widely used in plant studies. **Table 1** highlights that radioecologists have, mostly in a short time frame and under challenging conditions, generated significant data for some of these criteria. In the last decade, there have been calls for research that would, in effect, help fill radioecological gaps in the Bradford-Hill criteria, e.g., investigations of plant populations exposed to low doses of IR over a number of generations (e.g., Saghirzadeh et al., 2008), but few such studies have been reported or been focused explicitly on the relevant criteria. We emphasize that now data are available for some of the criteria in **Table 1**, future studies guided by the other criteria would be useful in determining how chronic low-dose IR affects plants over several generations.

**Table 1:** Preliminary assessment of data for observed effects on plants being caused by chronic low doses of ionizing radiation (IR) at about derived consideration reference level (DCRL) dose rates using the Bradford-Hill criteria.

Criterion	Preliminary assessment of data for effects of chronic low doses of IR in plants
l Strength	Several studies report statistical significance but the association between IR and effects is generally not strong. Use of meta-analysis to reveal effects indicate that they are weak. Possibility of selection bias in reporting of effects has not been analyzed.
2 Consistency	Different studies, different research groups, and research at different sites often produce conflicting evidence. Few studies have been repeated at the same sites. Truly blind studies reported.
3 Specificity	There is frequently the possibility of covariables in studies so effects cannot be securely ascribed to IR. In almost all instances ‘high’ dose rate sites vary in history of dose or in current environmental variables.
4 Temporality	There are few studies that describe endpoints before and after contamination so the relationship between the onset of elevated dose and effects is poorly known.
5 Dose-response	Some evidence for a dose-response but much debate about the relationship at chronic low doses.
6 Plausibility	DNA damage and repair well understood but cause of damage at low chronic doses unclear. Physiological explanations that effects are due to oxidative stress are implausible.
7 Coherence	The understanding of effects at different levels of biological understanding is not yet coherent.
8 Experimental	There is almost no experimental evidence derived under properly controlled conditions of effects of IR at chronic low doses over generations, years or decades.
9 Analogy	The actions of agents with similar effects, e.g., UV or ozone, at comparable exposures do not accord with purported effects of IR at chronic low doses.


Thus, overall, the frameworks used for radiological protection of the environment have more solid foundations, including those related to causality, at acute high doses and for humans and other animals than for plants. Here we use data about the effects IR on plants at all doses to provide a new synthesis that highlights, for protection of the environment, the importance of generating data under controlled conditions from multiple generations of plants growing at chronic low doses of IR.

## The Effects of Ionizing Radiation on Plants

The effects of IR in higher plants are of interest to agriculture, horticulture, ecology, and space science. We suggest that four particular aspects of plant biology provide a vital context for understanding the effects of IR. First, the light reactions of photosynthesis are initiated with photolysis of water – a processes with the same products as the radiolysis of water and that can result in the formation of enormous amounts of oxidative radicals that plants are generally able to disarm because of their high production of anti-oxidants (Willey, 2016). Second, in multicellular plants the dividing cells occur in meristematic tissues that have quiescent centers with functional equivalence to stem cells but that are not identical to them and do not have, for example, the same *p-53* mediated apoptotic capacity as animal stem cells. Meristems in plants are a biologically distinct product of an independent evolution of multicellularity (Fulcher and Sablowski, 2009) and the effects of IR on them are not well known. Third, the meiotic divisions that produce the gametophyte generation in reproductive organs in plants are separated in each generation by many vegetative cell divisions in the sporophyte generation – i.e., plants have an alternation of generations and no reserved germline. And fourth, although tumors can occur in plant tissues (Athena Aktipis et al., 2015), because of different controls on groups of multiplying plant cells (Doonan and Sablowski, 2010) and the reduced probability of metastasis in organisms without circulatory systems, plants do not suffer adverse cancerous effects of tumors to anything like the same extent as many animals. In plants there are, therefore, not likely to be the same stochastic effects of IR as in animals in which many such effects are cancers. Thus, current knowledge about the effects of IR on multicellular organisms is dominated by knowledge of effects on organisms with less anti-oxidant capacity than plants, that have stem cells and germ lines without exact plant equivalents, and that suffer stochastic effects unlikely to occur in plants.

### Molecular Biological Effects

#### Mutagenesis

The botanist Hugo de Vries introduced the concept of ‘mutation’ and suggested in 1904 that X-rays might induce them (Blakeslee, 1936). Thus, some of the earliest attempts at mutagenesis used plants exposed to X-rays and then radium (Stadler, 1928a,b, 1930). More than 2500 crop cultivars in current use, and that produce a significant proportion of all food consumed by humans, were developed using mutagenesis induced by acute high-dose IR (10 s of Gy or more) (Cheng et al., 2014). Purposeful IR-induced mutagenesis continues to play a significant role in the improvement of the world’s most important crops, e.g., rice and wheat, including through the use of ion-beams (Cheng et al., 2014; Zhang et al., 2016). The FAO/IAEA Mutant Variety Database registers numerous new cultivars each year, including many produced using IR. Such mutagenesis also has an important role in the development of new horticultural varieties (e.g., Taheri et al., 2014).

Oladosu et al. (2016) suggest that the changes in DNA during IR-induced mutagenesis can be of three sorts: (1) intragenic (point mutations within a gene sequence), (2) intergenic (inversions, deletions, duplications, translocations of DNA), and, (3) changes in chromosome number. Compared to other mutagens, IR can induce a relatively high incidence of DSBs in DNA. Mutagenesis experiments have frequently confirmed this with plants (e.g., Doná et al., 2013). High DSB incidence accords with the large deletions reported by Sato et al. (2006) and the many indels and copy number variations reported by Cheng et al. (2014) but single stranded breaks (SSBs) and other damage still occurs widely after IR exposure. For example, Cheng et al. (2014) analysis of Red-1 rice, a variety produced via IR mutagenesis, described altered sequences in approaching 9% of all genes primarily due to a rich variety of single nucleotide polymorphisms (SNPs). SNPs are not a simple product of strand breaks but of a plethora of differences, including in DNA repair systems. Further, the simple oxidation of bases can constitute 10–15% of all such DNA damage (Doná et al., 2013). Together with increases in alkali-labile sites, DNA–DNA, DNA-protein cross-links (Ventura et al., 2013), and long-known cytogenetic effects, such data show that acute high-dose IR can induce not just DSBs but essentially the full gamut of DNA damage in plants.

Mutagenesis experiments often note the capacity of plants to quickly repair a significant proportion of the damage caused by acute exposure to IR. For example, for horticultural breeding Taheri et al. (2014) note that *Curcuma alismatifolia* recovered significantly within 24 h from a 10 Gy dose and recommended that 20 Gy or more is necessary for useful net rates of mutagenesis. A frequent limitation to the use of comet assays in plant studies of the effects of IR on DNA is the significant capacity that plants have for DNA repair (Lanier et al., 2015). This is also a reminder that an acute external dose of IR is one of the mutagenic scenarios for which an acute dose is truly possible – in contrast to chemical mutagens that often continue to persist in a biological system after exposure has ceased. For example, in comet assays the ‘tails’ of damaged plant nuclei take longer to disappear after acute chemical exposure than after acute IR exposure (Ventura et al., 2013). Acute doses of 10–100 s Gy of IR are reported to produce ‘net’ rates of mutation from 10^-9^ base pair (bp) mutations per Gy (Fulcher and Sablowski, 2009) to 6.13 × 10^-6^ bp mutations per 500 Gy (Sato et al., 2006). Much radiological assessment of possible effects at low doses is based on extrapolation (using the linear-no threshold assumption) from observed effects at acute high doses down to low doses. In plants, such extrapolations, which assume a directly proportional relationship between dose and mutation rate, suggest that doses in the μGy and even mGy range will not induce mutation rates significantly above those that occur routinely in plants under field conditions. We suggest, however, that measurements of ‘net’ rates of mutation after acute exposure are unlikely to be appropriate for extrapolation to chronic exposures. With acute exposure, the period of DNA damage is essentially not contemporaneous with the subsequent period of repair, so comet assays in particular indicate that, even if it was appropriate to describe a ‘net’ mutation rate under these circumstances, the capacity for repair is sufficiently great that it is very practically challenging to measure effects with sufficient alacrity.

A variety of post-Chernobyl studies have suggested that chronic low-level irradiation of plants induces greater rates of mutation than predicted from acute high dose studies. Kovalchuk et al. (2000b), using wheat planted in soil contaminated from the Chernobyl NPP, suggested that a dose of 0.3 Gy over a growing season (assuming 100 days, i.e., 2400 h, gives dose rate of *c.*125 μGy h^-1^) produced a sixfold increase in mutation rate. Studies of *Arabidopsis* growing wild at Chernobyl have revealed that the incidence of genetic effects correlate with doses in contaminated areas (Abramov et al., 1992) as have a number of studies of Scots Pine (Geras’kin et al., 2016). The planting of previously unexposed populations of plants into contaminated soils at Chernobyl has suggested elevated mutation rates at dose rates around and sometimes below 100 μGy h^-1^. Overall, investigations of mutagenesis are a reminder of how high acute doses have to be in order to produce agriculturally or horticulturally useful mutation rates in plants, and also that the mutation rates reported at acute high doses are not particularly meaningful as ‘net’ rates because damage and repair are not occurring simultaneously. Although mutation rates reported in some chronic low doses studies are higher than predicted by extrapolation from high doses, and assuming the possibility of confounding factors in such studies can be discounted, it is still possible that such mutation rates are associated in some way with IR. Resolving this inconsistency between IR effects reported at acute high and surprisingly low chronic doses might be aided by an understanding of the processes of DNA repair in plants.

#### DNA Repair

DNA repair mechanisms that help reverse oxidative adducts and other chemical changes to DNA occur in higher plants, as does the induction of the cell cycle checkpoints necessary for the repair of strand breaks (Hu et al., 2016). Although the details of many of these processes are less well known in plants than in other organisms, it is clear that the mechanisms in plants for repairing strand breaks in particular are similar, though not identical, to those in other eukaryotes (Hu et al., 2016). In general, plant cells have greater resistance to the production of DSBs by IR and repair them more quickly than do animal cells, such that at a given dose they carry about 1/3 the DSBs that animal cells do (Yokota et al., 2005). This accords with known differences in radiosensitivity between plants and many animals and is likely a product of plant life strategies. Both the regular initiation of meristematic and reproductive tissue from vegetative cells and a sessile life-style that has to involve withstanding regular environmental insult from DNA-damaging agents, such Al^3+^ in acidic soils (Willey, 2016), promotes both a significant capacity for DNA repair and a resistance to the net effects of DNA damage. Interestingly, in contrast to multicellular animals, mutations of DSB repair proteins in plants tends just to reduce biomass production rather than change fundamental aspects of development (Manova and Gruszka, 2015) – emphasizing that the developmentally plastic modular growth form of plants provides a way not available to animals of resisting damage from mutagens.

In plants, the KU70/KU80 heterodimer recognizes DSBs, with *ku70* and *ku80* mutants being especially sensitive to DSB-inducing agents (Weimer et al., 2016). As in animals, in plants the MRN complex binds to DSBs and the RPA complex to SSBs, activating, respectively, the ATM and ATR pathways (Hu et al., 2016). In plants, ATM triggers the expression of SOG1 (Suppressor Of Gamma response 1) a transcription factor that acts as a key regulator of DNA repair processes (Yoshiyama, 2015). ATR acts through WEE1 to arrest the cell cycle and, probably through SOG1, to activate DNA repair. There are cyclins in plants that control cell cycle progression and the cyclin-dependent kinases (CDKs) that in turn control their activity. Cyclins are particularly functionally diverse in plants with, for example, CYCB1s and CDKB1s helping to regulate the repair of DSBs by either non-homologous end joining (NHEJ) or HR (Weimer et al., 2016). NHEJ, in which broken DNA strands are often simply ligated back together by LIG4 (DNA ligase 4) and XRCC4 (X-RAY REPAIR CROSS COMPLEMENTATION PROTEIN 4) (Bray and West, 2005), can result in altered DNA sequence if nucleotides are lost during breakage. At least three variations of the NHEJ pathways occur in plants (Charbonnel et al., 2011). Much HR repair uses a homologous chromatid as a template for high fidelity repair of DSBs and, therefore, occurs after the S phase of the cell cycle when chromatids have been duplicated but it can also occur between chromosomes or between homologous regions of a chromatid during the G1 phase of the cell cycle. Homology search, and strand incision, on sister chromatids is initiated by RAD51, of which there are five paralogs in plants. Weimer et al. (2016) suggest that a high level of redundancy in NHEJ and HR repair in plant cells contributes to, compared to animal cells, high resistance in plants to DSB-inducing agents. Redundancy in this context means not only a much greater capacity than is usually necessary but also a resilient capability based on multiple genes or pathways.

Acute high doses of IR to plants have been useful in elucidating the details of many of these DNA repair pathways but there are also reports of chronic low doses of IR inducing DNA repair – with studies of HR at Chernobyl, for example, providing a good example (Kovalchuk et al., 2000a, 2003). In general, based on conclusions drawn from mutagenesis studies, the sensitivity of induction of DNA repair in low dose/low dose rate IR regions is greater than predicted from that at acute high doses. It has also been shown that, at 100 mGy h^-1^ exposure, the radiosensitivity of plants to DNA damage declines with age (Biermans et al., 2015). The processes of DNA repair have evolved as a protective response to environmental insult and there are many other environmental variables that, at low intensity, cause an increase in the rate of DNA repair (Willey, 2016). Elevated rates of DNA repair at chronic low-dose rates are not necessarily detrimental even at the cellular level and it is certainly necessary to investigate their impacts at higher levels of biological organization to understand the biological significance of the effects.

### Gene Expression

Numerous authors have reported, in response to IR exposure, changes in gene expression in plants. In general, in experiments using high acute doses that have affected plant growth, the expression of 100 s, even 1000 s, of genes can change. This accords with the magnitude of changes in gene expression induced by, for example, growth change-inducing drought, temperature or salinity stress. No synthesis has yet emerged as to which particular pathways or processes are most effected by exposure to IR. Genes with altered expression generally include some that are involved in DNA repair and anti-oxidant defenses but also many others involved in a notable diversity of processes, including many Gene Ontology (GO) categories. For example, with acute doses of 100–2000 Gy over 24 h, Kim et al. (2014) revealed that the most numerous genes with changed expression had a role in; (a) catalytic activity, (b) the endomembrane system, and (c) active in metabolism. Hwang et al. (2014) using doses of 200 Gy gamma irradiation and 40 Gy Ion Beam irradiation over 24 h, noted gene expression related to sugar and starch metabolism were particularly affected. Published data include a notable proportion of reports of changes in flavonoid (e.g., Van Hoeck et al., 2017) and lignin metabolism (e.g., Lee et al., 2014). Park et al. (2015) investigated the radiation responsive *OsGIRP1* gene at 100–400 Gy in rice, revealing that its expression helped to control the degradation of key photosynthetic proteins that might have been damaged by high dose IR.

Kovalchuk et al. (2007) revealed differences in gene expression in *Arabidopsis thaliana* exposed to a total of 1.0 Gy as an acute dose (delivered at 90 Gy h^-1^) and as a chronic dose (21 days at 1.8 mGy h^-1^). The acutely exposed plants demonstrated up-regulation of genes involved with DNA repair, oxidative stress response and signal transduction pathways, whilst the chronically exposed plants showed no alteration of expression profiles of genes associated with DNA repair or cell antioxidant response. Goh et al. (2014) demonstrated a similar effect at 200 Gy, which induced changes in gene expression when spread over hours but when spread over 2 or 3 weeks had no effect on gene expression. There is also evidence that the stage of plant development affects both the expression of IR sensitive genes in unexposed plants and their general plant response to IR (Biermans et al., 2015). Kimura et al. (2008) studied gene expression changes on rice seedling leaves post low-dose exposure to IR from contaminated soil in the Chernobyl vicinity. Experiments showed that >500 genes responded to radiation. Up-regulated genes were associated with cellular processes and signaling actions to specifically include defense, cell wall synthesis, and secondary metabolite biosynthesis. Down-regulated genes indicated suppression of information and storage functions alongside non-specific metabolic pathways. Sahr et al. (2005) reported changes in the expression of 46 genes involved in fundamental cellular processes in the roots of *Arabidopsis* from internal dose rates of 50–100 μGy h^-1^ from ^134^Cs. It seems clear, therefore, that not only high acute doses but also quite low chronic doses of radiation can affect gene expression in plants. Although the pathways and processes particularly effected are not clear, there are indications that over time the effect on gene expression at chronic low doses might attenuate. It should, however, be noted that genomic technologies are sensitive, so changed expression in a few tens of genes out of many thousands does not necessarily lead to adverse effects at higher levels of biological organization.

#### Effects on Plant Proteomes and Metabolomes

Acute high doses of IR to plants change their protein and metabolite profiles. For example, Roitinger et al. (2015) using *atm* and *atr* mutants reported changes in ATM- and ATR-dependent pathways and in phosphorylation patterns of the proteome. In rice varieties produced by IR mutagenesis Hwang et al. (2015) report, for example, changed carbohydrate and protein degradation metabolism. Often such changes include those to anti-oxidant systems (Goh et al., 2014; Ramabulana et al., 2015). Several studies have shown that γ-radiation has an effect on chlorophyll content. Alikamanoǧlu et al. (2007) found that total chlorophyll content (from both chlorophyll *a* and *b*) increased when *Paulownia tomentosa* was exposed to 5–50 Gy at a rate of 10 Gy h^-1^. Chlorophyll content was also significantly increased in red pepper (*Capsicum annum*) at 16 Gy (Kim et al., 2011). As with changes reported in gene expression during high-dose exposure, such data suggest that; (1) the magnitude of changes in proteomes and metabolomes is as expected from severe environmental stress, (2) what might differentiate IR-induced changes from those induced by other stressors is not yet clear, and (3) that few changes might be expected at much lower doses.

At Chernobyl, however, differences in seed proteins, have been reported between contaminated and control plots (Klubicová et al., 2012; Rashydov and Hajduch, 2015), and Hayashi et al. (2015) reported changes in rice proteomes at ‘ultra-low’ level gamma doses. In Chernobyl seed studies, contamination levels of *c.* 20 kBq^137^Cs/kg soil plus *c.* 5 kBq^90^Sr/kg soil probably give doses to plants of no more than 100 μGy/h (though doses to plant parts might be different depending on internal accumulation of radioisotopes), whilst in the aforementioned Fukushima studies (Hayashi et al., 2015) doses were a maximum of 4 μGy/h. Overall, proteomic data from field experiments on seeds at Chernobyl showed that plants growing in the zone responded similarly in their proteome to plants undergoing stress from heavy metals (Danchenko et al., 2009). Thus, there is evidence of effects of IR on the proteome and metabolome at low chronic doses. However, again it must be remembered that many of the ‘omics’ techniques are very sensitive, and that sessile plants experience the environment as constantly changing and are, therefore, constantly responding to it. For example, plants respond daily to the day/night light cycle with profound changes in their proteomes and metabolomes. It is, therefore, vital to assess not just whether changes in genotypes, proteomes and metabolomes can be detected but whether these chronic low-dose induced changes translate into significant changes in phenotypes because it is, primarily, phenotypes that determine the fitness of individuals and populations in the environment.

### Effects of IR at Whole Plant Level

#### Shoots

Acute high external doses of IR have long been known to affect most aspects of shoot growth, with recent reports including effects on developmental timings (Nishiguchi et al., 2012; Sidler et al., 2015), morphology (Celik et al., 2014; Sever-Mutlu et al., 2015), anatomy (De Micco et al., 2014), and the development of bulbs (Mostafa et al., 2015). As there have been for many years, there are recent reports that acute high doses, mostly to propagules, sometimes have positive as well as negative effects on subsequent growth. For example, at 10 Gy given over 10 s Hamideldin and Hussien (2014), using different potato varieties, noted some positive as well as negative effects on subsequent height, leaf area, stem diameter, and tuber diameter. Several studies carried out in the immediate aftermath of the Chernobyl accident not only confirmed the sensitivity of the shoots of some species to IR and but also detailed a variety of effects that supplemented significantly knowledge about acute effects of IR in the field. These studies have now been complemented by some research to elucidate the effects of chronic low doses.

Mousseau et al. (2013) using wood cores of *Pinus sylvestris* at Chernobyl provided evidence that trees in locations near the reactor had different, and more variable, growth rates of above ground parts after irradiation from the accident. Although these effects were correlated with dose rates in 2009, it was not possible to disentangle the effects of high acute post-accident doses from any due to subsequent lower doses. At Fukushima, studies of *Abies firma* growth from before and after the accident also found effects on growth that, although they correlated with dose rate in 2015 were not necessarily produced by it (Watanabe et al., 2015). The extensive studies carried out on *P. sylvestris* in the Bryansk region of Russia since 2003, and that include detailed dose calculations, can more clearly distinguish effects caused by chronic low doses in the period remote from the accident. In general, these studies ‘are consistent with an international recommendation to consider radiation exposure of 100 mGy/a (c. 10 μGy/h) as a margin for biota safety in chronic irradiation’ (Makarenko et al., 2016). However, recent studies at this location have supported the assertion that *P. sylvestris* is particularly sensitive to IR. They have noted an increased frequency of gene mutations at 1.14 μGy/h (10 mGy/a – below the low end of the DCRL for *P. sylvestris*) and changes in anti-oxidant concentrations at 5.7 μGy/h (50 mG/a – just in the range of DCRL for *P. sylvestris*) (Volkova et al., 2017). It is notable that of the many endpoints measured in these studies, there are some in which significant effects of IR are reported, especially cytogenetic ones, but that these are not, overall, adverse enough at the level of the individual or above to merit a reconsideration of the DCRLs. At the Semipalatinsk nuclear test site in Kazakhstan, studies of *Koeleria gracilis* (crested hair grass) that had inhabited for 50 years soils contaminated with radioactivity and with a current dose rate of 4–285 mGy/a, also showed cytogenetic effects at the highest doses but no morphological effects (Geras’kin et al., 2012). Seeds collected from the most exposed plants did not differ in their response to irradiation suggesting that IR has not exerted any selection pressure over 50 years and that recommended dose limits were appropriate.

Studies carried out near Chernobyl have also provided evidence of effects on whole plants at chronic low doses. In studies on *P. sylvestris* planted after the accident at Chernobyl and investigated 25 years later, normalized dose rates for the period, based on the sum of both internal and external doses, of 10 μG/h and less were related to significant cytogenetic and morphological effects (Yoschenko et al., 2011). At 40 G/h there were significant effects on apical dominance, with cytogenetic effects being related to incidence of morphoses. However, in experiments with *Lemna minor*, which enables detailed developmental analysis under controlled conditions, doses of 80 μG/h to 4.95 mGy/h had no effect on physiological, morphological, or developmental parameters (Van Hoeck et al., 2015). Overall, therefore, some effects of chronic low dose IR on individual plants shoots have been reported at the low end of DCRL ranges but it has not been suggested that they are significant at the population or community level.

#### Roots

Plants are well known to respond to soil stresses via changes in their roots (e.g., Bochicchio et al., 2015), which can then affect overall plant function. Gunckel (1956) noted that roots are shielded from much α and some β IR by the soil which, together with practical difficulties of experimenting with roots, may have contributed to relatively few studies of the effects of IR on roots having been reported. However, the fact that the long-term fate of much contamination following accidents at Kyshtym, Chernobyl, and Fukushima has been soil root zones highlights how important the effects of IR on roots might be. This is particularly relevant in the earliest stages in the plant life-cycle that have particular proximity to the soil and that are generally the most susceptible to the effects of stress. Further, even for the biologically mobile Cs, accumulation from root uptake is almost always higher in roots than shoots (Danchenko et al., 2016) – a distribution that is generally more pronounced the less mobile a radioisotope is.

Acute high doses of IR have long been known to quickly affect roots, primarily via the root meristem. Gray and Scholes (1951) found that irradiated *Vicia faba* roots (1.2 Gy) had inhibited growth and that exposing only root meristems had the same effect as exposing the entire root system. In pea and maize, survival of root apical meristems post-irradiation event (3–32 Gy) showed that radioresistance at different points in the cell cycle varied slightly between species, and that there were overall differences in resistance depending on phases of early growth (Gudkov and Grodzinsky, 1982). Duration of individual phases of the cell cycle and overall cell cycle period was also changed depending on species. Exposing *Arabidopsis* roots to 3 kGy inhibited elongation from the root tip and induced root hair elongation and cell expansion (Nagata et al., 2004). Some studies report either root elongation or growth inhibition depending on dose (Maity et al., 2005; Yadav, 2016). Acute doses from ion beams on root meristems indicate that they are a key exposure site (Zhang et al., 2016) and several studies note the role of changes in ROS in roots after acute high exposures (e.g., Nagata et al., 2004).

Biermans et al. (2015) using solution cultures reported that, over 7 days, doses of 11 mGy/h from ^241^Am reduced the root growth of *Arabidopsis* and affected its dry matter but that lower doses did not. Sahr et al. (2005) reported that dose rates of 100 μGy/h (from 60 kBq/L ^134^Cs in a solution culture) affected *Arabidopsis* root growth but that doses of 50 μGy/h did not. Below these dose rates there are no reports of morphological changes, although several studies have reported genetic and cytogenetic changes. A standard *Allium* root tip test revealed a linear relationship between dose and chromosome aberrations up to a dose of about 80 μGy/h in Chernobyl contaminated soil (Kovalchuk I. et al., 1998; Kovalchuk O. et al., 1998). Similar studies with ^90^Sr contaminated sites have also shown similar effects at even lower dose rates. In naturally enhanced background areas at Ramsar (with up to 12,500 Bq ^226^Ra/kg soil and doses of up to 100 μGy/h) Saghirzadeh et al. (2008) also described chromosomal aberrations in *Allium* root tips. However, in neither of these studies were threshold relationships tested.

There is, therefore, much to be learned about the effects of IR on the ‘hidden half’ of plants. It seems likely that there are detectable effects of chronic low doses at the genetic and cytogenetic levels at the low end of DRCLs, and perhaps below. There is some evidence of morphological, or other whole root effects, close to DCRLs. Downie et al. (2015) emphasized how often roots are examined artificially flat and that there is still a lack of focus on root-environment interactions. Methods for examining roots *in situ* have been developed for a variety of media including soil (Yuan et al., 2016), paper wick (Adu et al., 2014), and gels (Bochicchio et al., 2015), which would be very useful for examining the effects of chronic low dose IR on root systems.

Overall, plant morphology has long been known to alter when exposed to high doses of radiation. In recent years, advances in image-based analysis has enabled the study of phenomics. Phenomics is concerned with phenotypic variation and its causes, effects, and implications. Houle et al. (2010) explained that understanding of phenomics is far less comprehensive than that of genomics, and we suggest that the same can be said to an even larger extent within the field of radioecology. Morphometrics, the quantitative analysis of shape and/or form of a subject is fast-becoming a key method of producing high-throughput data for phenomics. We suggest root and shoot studies in radioecology should employ high throughput image analysis to complement the increasing plant stress biology phenomic data – it is a powerful way of analyzing subtle environmentally induced changes in plants.

### Reproductive and Transgenerational Effects

Reproductive organs are often especially sensitive to the effects of environmental stress, with potential implications at the community and population level. Thus, in *Caenorhabditis elegans* investigations of the impact of IR often use reproductive end points (Buisset-Goussen et al., 2014). In general, propagules in plants almost always have very high, often extremely high, levels of redundancy, i.e., the toll of adverse environmental effects (which essentially always exist in the wild) on success is overcome by the high numbers produced. There are many reports that acute exposure of seeds to high dose rates of IR produce hormetic effects on subsequent growth (recently, e.g., Maity et al., 2009; Marcu et al., 2013a,b; Ahuja et al., 2014; Yadav et al., 2015). The effects are generally short-term and the role of, for example, heating or commensal micro-organisms in producing the effects is unknown. Acute high dose rates have also been shown to affect a variety of seed constituents (e.g., Jan et al., 2012; Tilaki et al., 2015; Vaizogullar and Kara, 2016), which might affect subsequent germination and growth. In the field, soon after the Chernobyl NPP accident, dose rates around 2 mGy/h produced lethal embryo mutations in *A. thaliana* (Abramov et al., 1992) and extensive studies of *P. sylvestris* near the Chernobyl NPP have shown that plants that received total doses of >2 Gy in areas of high short-term contamination had decreased reproductive ability and that this effect lasted for more than a decade (Fedotov et al., 2006). Boubriak et al. (2008) reported in pollen collected from control and contaminated sites near the Chernobyl NPP different IR exposure affected the rate of DNA synthesis. In general, seeds and pollen have high resistance to environmental stressors but, perhaps because some IR can penetrate their protective coats, it seems that relatively low total doses delivered at high dose rates can have effects, including hormetic effects, whilst large doses received at high dose rates produce significant adverse effects.

Based on studies with 94 species (Kordium and Sidorenko, 1997) and 111 species (Møller et al., 2016), it has been suggested that, at time periods remote from the high post-accident doses, in the area around the Chernobyl NPP about 10% of species have slightly decreased pollen viability associated with enhanced doses of IR. Møller et al.’s (2016) study was carried out in 2008–2011 and included maximum dose rates of about 150 μGy/h. In long-term studies of *P. sylvestris* in the Chernobyl-contaminated Bryansk Oblast of Russia, germinating seeds have rates of cytogenetic damage of up to 1.3% that correlate with dose rate (Geras’kin et al., 2011), and that is repeated elsewhere at even lower dose rates (Evseeva et al., 2011). Several detailed studies of plants growing in the East Urals Radioactive Trace, which has the longest history (1957 onwards) of any widely studied radioactively contaminated site and has dose rates of up to 240 mGy/y (*c.* 28 μGy/h), have shown dose-dependent effects on germination or viability of seeds of *Taraxacum officinale* (Pozolotina et al., 2012), *Melandrium album* (Antonova et al., 2013), and *Leonurus quinquelobatus* (Karimullina et al., 2015). Several authors have noted that chronic low dose rates of IR can make germination more variable, particularly in response to weather conditions (Antonova et al., 2013; Geras’kin et al., 2016) and other soil contaminants (Evseeva et al., 2009; Karimullina et al., 2015). There is, however, evidence from studies in Bryansk, Russia, that such effects do not alter the overall reproductive capacity of *P. sylvestris* (Geras’kin et al., 2016).

Studies in areas contaminated from the Chernobyl NPP accident (in particular with the relatively sensitive *P. sylvestris*), and especially in the EURT, have shown that chronic low dose effects on plant propagules can be sustained for many generations. Boratyński et al. (2016) hypothesized that effects of IR on life history responses might be sustained for generations in the absence of irradiation. Wild carrot plants, sampled from around Chernobyl (0.08–30.2 μGy/h) and then grown in uncontaminated soils in a greenhouse showed correlations between previous radiation dose and the timing of developmental events. The presence of trans-generational effects has perhaps helped prompt some discussion about ‘adaptation’ of plants to chronic low-level doses of IR. For example, studies of flax and soya seeds grown over several generations near the Chernobyl NPP have shown differences in seed constituents and prompted suggestions of adaptation to chronic low dose IR (Gabrisova et al., 2016 and references therein), as have effects of high doses on pollen (Boubriak et al., 2008), the ability of plants from Chernobyl to resist the effects of mutagens (Kovalchuk et al., 2004) and studies at a number of other contaminated sites (e.g., Geras’kin et al., 2013; Møller and Mousseau, 2015; Boubriak et al., 2016). These references, and references therein, provide evidence that at chronic low doses in the range of a few 10 s of μGy/h, some plants can have increased heterozygosity, increased rates of DNA repair, and increased variability of key seed properties and constituents. There is also evidence of some increase in radioresistance, at the DNA and cytogenetic level, in some species at these dose rates. We suggest that great care has to be used in interpreting these effects as ‘adaptation.’ An adaptation increases the fitness of an organism, i.e., its ability to survive under conditions of natural selection (Futuyama, 2009). No data that we are aware of has actually demonstrated this to be the case for plants exposed to chronic low levels of IR. However, an increase in diversity of many phenotypes is common under other stress conditions and, in some instances, has been shown to provoke the evolution of an adaptation to them.

There have also been some investigations of epigenetic effects of chronic low-dose IR on plants. Epigenetic effects are inheritable changes in phenotypes that cannot be explained by changes at the genetic level (Waddington, 1942; Weinhold, 2006). They occur because of, for example, heritable changes in methylation of DNA (which effects gene expression rather than sequence) or changes in histones (which control DNA packing and unpacking). Exposure to stressors has the potential to reshape not just the genome but also the epigenome, changes to the later probably being quite common in organisms (Grossniklaus et al., 2013). There is, however, some debate about how significant these effects might be in the long-term in plants (Pecinka and Scheid, 2012), in part because of the higher basal rates of methylation in plant DNA than animal DNA. Nevertheless, epigenetic changes can be important in plants. In the halophytic species *Mesembryanthemum crystallinum*, under drought conditions the plant has the ability to switch metabolic pathways from C_3_-photosynthesis to Crassulacean Acid Metabolism (CAM). This change of pathway involves profound changes in the control of, for example, stomata function and enzyme activity, and is mediated to changes in DNA methylation (Dyachenko et al., 2006). High doses of radiation (10 Gy) that effect plant development of 20 days old plants change the expression of enzymes that mediate DNA methylation (Sidler et al., 2015) and *P. sylvestris* trees exposed to high doses post-Chernobyl have hyper-methylated DNA (Kovalchuk et al., 2003). Germinating soya bean seeds from plants grown in Chernobyl-contaminated soil for six generations produced rootlets with slightly enhanced levels of DNA methylation (Georgieva et al., 2017).

Overall, chronic low doses (in a range as low as 5–50 μG/h) have been reported to have detectable effects on plant seeds. The data on which this assertion is made is primarily field based and, given that there is some evidence that effects can be sustained through the generations, an assumption that current exposure to IR explains currently observed effects must, in a number of instances at least, come with the usual caveats about confounding variables in field studies. It must also be noted that the effects tend to be of low frequency in a structure that is generally produced with a high level of redundancy. There is, overall, little real evidence of any adaptation to chronic low-level IR across generations (Møller and Mousseau, 2016) and we suggest that if it occurred to a significant extent it would have been more securely established in field studies – many plant species can adapt quickly and obviously to, for example, the presence of inorganic and organic contaminants in soils (Willey, 2016). In addition, environmental variables such as temperature or water availability frequently have significant, often catastrophic, effects on the production or viability of seed in any given year without necessarily affecting populations in the long-term. Against such a background, the significance of some low-incidence effects on reproductive propagules just below the DCRL range needs to be assessed at the population and community level but seems unlikely to be significant to populations in natural ecosystems.

### Effects on Plant Populations and Communities

Key insights into plant population biology and community ecology have been derived from studies of stress and disturbance. From early on in the nuclear age, high dose IR of 10–100 s of Gy was used not just to understand its effects but also to gain fundamental ecological insights using its unique properties as a stressor – high activity point sources produced predictable, continuous gradients of stress and could be switched on and off using shielding. For example, the United States Atomic Energy Commission’s experiments, primarily in the 1960s, with high activity point sources in a variety of ecosystems (Jordan, 1986) informed early thinking about tropical forests in particular (Lugo, 2004) and the results of studies at US nuclear weapons test sites in Micronesia probably influenced important conceptions of ecosystem ecology (DeLoughrey, 2013). Aside from ecological insights, from these studies, and from those in the USSR, it became clear that populations of plants were most sensitive in the order trees > shrubs > herbs, and that coniferous trees were more sensitive than hardwood trees. It was originally suggested that sensitivity of plant populations correlated with chromosome size and number (e.g., Woodwell, 1962) but later syntheses of these experiments suggest a better correlation with proportion of non-photosynthetic to photosynthetic material (Jordan, 1986). Plant populations that were killed by massive doses close to point sources had, when studied, not recovered decades later (Stalter and Kincaid, 2009) but plants more distant from sources helped inform the early IAEA suggestion that a dose rate of 100 μGy/h or less did not affect plant populations.

Numerous studies post-Chernobyl in locations proximal to the reactor that received high acute doses added an impressive range of details to the understanding of high dose effects and, overall, supported previous suggestions about the adverse effects of high doses and of the sensitivity of plant populations. In particular, *P. sylvestris* was found to be particularly sensitive and *Picea abies* even more so (Geras’kin et al., 2008). At sites contaminated from the Chernobyl accident together with other studies in Russia in the EURT and at U-mine tailings, lower dose rates (even at around previously suggested dose limits) have shown cytogenetic effects (Geras’kin et al., 2013), decreasing significantly the dose rates at which effects have been demonstrated. The significance of these effects for plant population health is unclear – at U-mine tailing sites there is the possibility of chemical toxicity explaining some of the effects that might change populations and at Chernobyl-contaminated sites the possibility of persistence of effects from previous high dose exposure to populations might do so. At the Semipalatinsk test site, there is good evidence of cytogenetic changes at doses of 10 s μGy/h but also good evidence that it does not affect plant populations (Geras’kin et al., 2013). Climate, soil type, species of plant, and the topographical and geological features of a region all affect the behavior and effects of IR in natural ecosystems. Research on the dynamics and effects of forest contamination in the long-term is still vital because, even though more than 30 years have passed since the Chernobyl accident, such a time period is only half of an average forest cropping cycle in many contaminated areas (Takahashi et al., 2016). Overall, the evidence suggests that the cytogenetic changes found in the DCRL range probably do not affect population characteristics or that if they do the effects are subtle. Subtle effects may be of some ecological significance, with the magnitude of stress and disturbance from other sources perhaps playing a key confounding role.

## Plant Biology and Ionizing Radiation – a Stress Response Context

**FIGURE 6:**
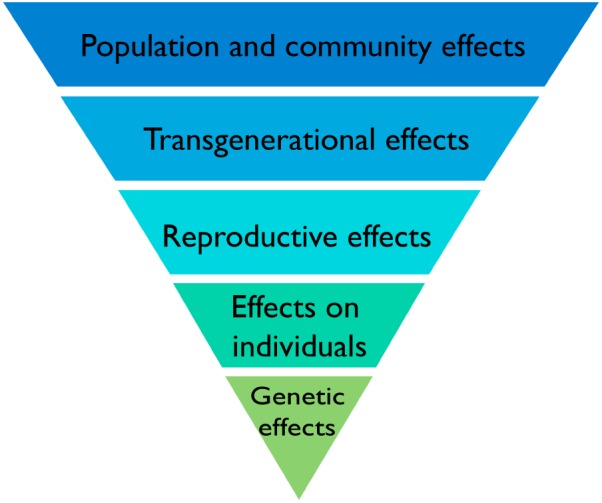
A biological hierarchy of effects in response to IR. The biological level at which effects of IR are described is important because there is not necessarily a direct relationship between effects at different levels. Increased mutation rates in DNA are commonly induced by IR but do not necessarily translate directly into effects on individuals or on individual fitness because plants have the capacity to repair them. If effects do, however, occur in individuals they do not necessarily affect their reproductive fitness or if they do, do not necessarily affect the functioning of communities and ecosystems. Protection of the environment from the effects of IR focuses on protecting biodiversity, populations, communities, and ecosystems and it is effects at these levels that are of significance rather than the detection of particular effects at the genetic level.

The land surface is a challenging environment for life, not only because some of life’s essential resources (e.g., water and nutrients) can be in short supply but also because terrestrial environments tend to be more variable, both spatially and temporally, than the aquatic environment in which life originated. It was many years after it evolved that multicellular life adapted to the challenges of life on land, as evidenced by the relatively late colonization of the land surface by plants approximately 450 Ma ago. Numerous aspects of the biology of terrestrial plants are a product of the challenges of life on land. A biological hierarchy of effects that such challenges provoke can be used to visualize this (**Figure 6**). It is within such stress-response perspectives that the responses of terrestrial plants to IR might most fruitfully be viewed.

Higher plant shoots, for example, are adapted, often from molecular to population scales, to capture light, exchange gasses and transpire water – reflecting the key sources of stress to terrestrial plants. Taking these in turn, the land surface is often bathed in more light than plants need and photoinhibition of photosynthesis is common, even after the evolution of many adaptations to control it (Raven, 2011). Photoinhibition results from loss of control over photolysis – the splitting of water in photosystem II (PSII) – producing ROS that damage photosynthetic machinery. Plants are adapted, at various biological levels and to different extents depending on their environment, to both minimize the production of oxidizing radicals that routinely leak from PSII and to nullify their effects as much as possible. Photoinhibition occurs when, relative to those produced from radiolysis at even medium dose rates, very high amounts of oxidizing radicals are produced. Photoinhibition is common not just in high light environments but also in northern latitudes when it is cold (Takahashi and Murata, 2008). Many of the gasses that plants can be exposed to, naturally and as pollutants (e.g., CO_2_, SO_2_, NO_x_, and O_3_) enter through stomata and dissolve first in the apoplastic solution, which causes, amongst other stresses, redox control challenges that can result in the production of ROS (Shapiguzov et al., 2012). When fresh water is in short supply, which is a very important stress to higher plants on land (Claeys and Inze, 2013), cellular redox balance is disturbed and unusual concentrations of ROS occur in cells. The production of ROS induced by these, and other, abiotic stressors adds to those that routinely leak out of mitochondria and those produced in response to a variety of biotic attacks.

There have been numerous reports of changes in anti-oxidant concentrations due to chronic exposure to low-dose IR (often with reports of oxidative ‘stress’) (Einor et al., 2016; Volkova et al., 2017), and some studies consider that the ‘dominating effect of IR in cells is the formation of free radicals from water or oxygen’ (Danchenko et al., 2016), but we suggest that these claims must be considered within an appropriate stress response context for higher plants. Not just in plants but in any aerobic organism, the disturbance of the delicate redox balance of life, rather than a change in anti-oxidant concentration, underpins oxidative stress. In plants ‘stress’ is usually defined as, for example, ‘any unfavorable condition or substance that affects or blocks a plant’s metabolism, growth, or development’ (Lichtenthaler, 1998). Anti-oxidants exist to buffer the redox poise of a cell against change and a change in their concentration does not necessarily show that redox poise has been changed or that normal metabolism, growth, or development has been blocked. And stress produces radicals that are both a cause and a consequence of ‘stress’ – so changes in anti-oxidant concentrations might be a consequence of other damage rather than direct oxidative stress. The radicals produced by low doses of chronic IR even in contaminated environments are few compared to those produced routinely by life processes and by other stressors, and plants have adapted to deal with them at a full range of biological scales. It is possible that IR’s penetrating power, compared to UV for example, and its uncompartmentalized production of radicals, compared for example to those produced in plastids, is particularly challenging to life but we suggest that the reported changes in anti-oxidant concentrations should not be used as evidence that IR in currently contaminated environments is directly causing oxidative stress in plants, especially at the population and community level, and compared to that from other sources. Given the significant anti-oxidant capacities of higher plants and their adaptation at a variety of biological scales to oxidative challenge produced by variation in many other environmental variables, it seems unlikely that there will ever be evidence for biologically significant direct oxidative stress to plants from low-dose chronic IR.

**FIGURE 7:**
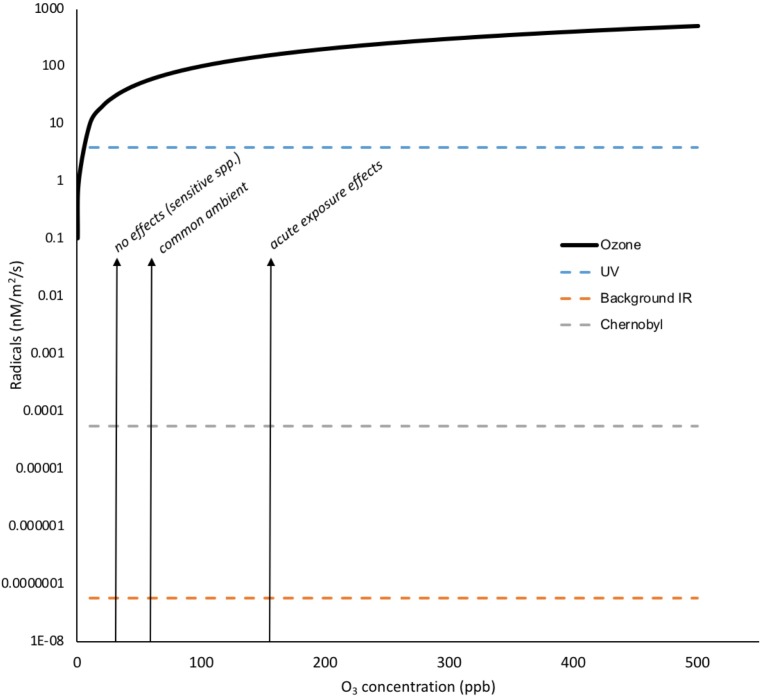
Oxidizing radicals from O_3_ exposure compared to those from background IR, Chernobyl and UV. Concentrations of tens of ppb O_3_ have documented effects on plants and concentrations of >150 ppb can have visible effects. To calculate the total radical production by O_3_ in nM/m^2^/s its partial pressures were calculated in Pa for the 0–500 ppb range and, using the ideal gas equation (PV = nRT), the molarity in air was calculated. Using an Oswaldt’s constant (*H*) of 0.25 for O_3_ in air/water mixture (effectively an air/water distribution coefficient that is equivalent to Henry’s law constant) and the relationship molarity in air = *H* × molarity in water, the molarity of O_3_ dissolved to water at the appropriate range of partial pressures was calculated. To calculate radicals in nM/m^2^/s we assumed ten thousand liters of air per m^2^ (i.e., a relatively shallow depth of air), an effectively infinite supply of O_3_ and that every O_3_ molecule produced a radical. The dissolution of O_3_ to water is affected by numerous factors including temperature, solutes, the presence of anti-oxidants and so on but it is clear that the number of oxidizing radicals produced by IR at Chernobyl are several orders of magnitude less than the concentrations of O_3_ deemed to have no oxidative effects on plants. (Details of calculations are given in **Supplementary Data Sheet S5**).

When it occurs, chronic oxidative challenge can, of course, have significant effects on plants – tropospheric O_3_ contamination is estimated, through oxidative effects, to decrease global production of staple crops by 3–12%, which equates to 10 s of $bi per year lost production (Van Dingenen et al., 2009). The ‘O_3_ equivalent’ of IR exposure provides a revealing comparison for the chronic long-term oxidative effects of IR. A preliminary comparison (**Figure 7**) suggests that activities of environmental IR not just at, for example, DCRLs but also some orders of magnitude above, will produce many fewer ROS than ambient O_3_ concentrations and that increases in O_3_, which are occurring in many parts of the Earth’s terrestrial surface, are likely to be much more oxidatively challenging to plants than low-dose chronic IR. Given the stress response context for higher land plants and the established production of so many ROS under so many conditions it is, we suggest, difficult to see how chronic low dose exposure to IR at DCRLs, and perhaps a magnitude above at least, adds significantly to ‘stress’ from oxidizing radicals. This might also prompt more thought about claims of ROS and anti-oxidant capacity being important for the effects on animals of chronic exposure to low doses of IR.

Thus, if there are effects of chronic exposure to low dose IR at the lower end of the DCRL range or below, we suggest that a stress response context indicates that they will be primarily as a result of DNA damage rather than ‘oxidative stress.’ The life strategy of terrestrial plants involves coping with stress using high cellular capacity for DNA repair, especially DSBs, the development of reproductive structures *de novo* in each generation rather than from a dedicated germline (which helps limit the multi-generation effect of deleterious mutations), and high redundancy of reproductive structures during reproduction. Overall, the effects of IR on plant populations at chronic low doses, if or when they occur, are likely due to DNA damage and can be expected to occur at doses that are higher than in more IR sensitive organisms. Given that plants are adapted to a hierarchy of effects induced by stress (Willey, 2016), and that this adaptation is often based on processes with a high level of redundancy, it is clearly possible that effects detected in cells or individuals do not manifest as biologically significant effects at the population or community level.

## Discussion and Conclusion

Particular consideration of the effects of chronic low-dose IR on plants is necessary because of the predominance in the literature of data derived from acute high doses to organisms with a different biology plus a lack of data from plants experiencing chronic low doses under controlled conditions. Field data following nuclear accidents is vital not only for managing contaminated sites but also because it provides insights into the effects of chronic low-dose IR under conditions in which; other abiotic and biotic stressors are present, competition is likely to be occurring, complex ecosystem interactions are present, and in which exposure can include emissions from hot particles (Sandalls et al., 1993). More data on the effects of low dose IR under controlled conditions might help clarify not only its effects but also help to identify the role of interactions with other variables in producing effects of IR observed in the field. However, in general, currently available data collected over several decades suggests that: (a) biological contexts are important when trying to understand effects across a range of doses and at different levels of biological organization; (b) at the sub-cellular level, low dose chronic IR can have detectable effects on plants primarily via minor changes to genetic material; and, (c) that in DCRL dose ranges these effects do not have significant adverse consequences for plant populations and communities. Thus, overall, we conclude that existing evidence in appropriate context suggests that current environmental protection frameworks for flora are generally fit for purpose. The sessile life strategy of plants and the static icon of the DNA double helix can be distracting. The survival of individual plants, and their populations and communities, is based on a life strategy which, although it does not involve much individual mobility, uses dynamic processes at a wide range of biological scales that often have high levels of redundancy.

A situation in which effects might be detectable but a fundamental change in environmental protection frameworks is unnecessary is not necessarily paradoxical and arises from, and is acceptable under, the following circumstances. First, the available data. Much relevant data from field studies is available and, although it was often not designed, for good reason, to test dose limits, only some of the data indicate effects at the low end of DCRLs and often for plants long-known to be sensitive to IR. Further, in data that include effects at these levels the evidence for causality is often associative and meets only some of the Bradford-Hill criteria. Thus, especially when many data sets do not report significant effects at relevant dose rates, there is not a conclusive enough body of evidence to change, for example, DCRLs for plants. Second, the type of effects reported. Where effects have been reported at DCRLs or sometimes below, they are sub-cellular and there is no real body of evidence of effects at higher levels of biological organization. DCRLs, and other frameworks for radiological protection of flora and fauna, aim to protect biodiversity, conserve species and protect the health of communities and ecosystems – which the evidence suggests that they do. Third, the biological context. Sessile life on the land surface is stressful and plants have evolved to cope with high levels of variation in their environment. They do this in part using DNA repair mechanisms and anti-oxidant pathways that have a higher capacity than many other multicellular organisms and that cope routinely with stress more than equivalent in magnitude to that from chronic low dose IR at DCRLs. Just because an environmental variable causes a change in a cell it does not mean that the cell, or the individual it is part of, is stressed or that it will necessarily be adversely affected. During the evolution of plants average dose rates probably peaked at 20 μGy d^-1^ and it seems sensible to suggest that high levels of DNA repair and anti-oxidant activity in plants prompted by other stressors enable them to generally suffer no adverse consequences of IR, at the population and community level at least, up to the low end of the DCRL range for sensitive plants (4 μGy h^-1^). It is, however, notable that the species for which IR effects data is available constitute a very small proportion of the world’s plant species so there is likely to be room for improving our understanding of which species in particular are protected by the grass and pine tree RAPs. It is possible that there are plant species, perhaps those that have specialized in living in particular conditions, that are especially sensitive or resistant to the effects of IR and that merit their own DCRLs.

Investigating data on the effects of plants at a range of exposures provides some directions for further research. First, it emphasizes the importance of the particular biological context. More data on the effects of IR, especially low-dose chronic IR, on roots, meristems, plant reproductive structures, and plant developmental endpoints over multiple generations seem especially important. Second, it emphasizes the plant stress-response context. Plants are adapted at a hierarchy of biological scales to resist significant environmental stressors, including many that act via mechanisms similar to those through which IR acts, so the existence of effects at a sub-cellular level should not be viewed as necessarily having adverse consequences at higher levels of biological organization. Third, IR is a primordial stressor. At a time in Earth’s history when unprecedented anthropogenic environmental changes are occurring and plant responses to these changes are vital to global food supplies and ecosystem functioning, understanding the effects of IR on plants might also be useful for understanding the evolution of plant stress responses in general. Finally, it emphasizes the societal context of the protection afforded to the environment. If the protection of biodiversity, communities, and ecosystems from the effects of IR is the goal then the evidence suggests that current systems are appropriate. If, however, as is the case with protection of humans from the effects of carcinogens, protection of individuals from rare stochastic effects is important, then it is possible that between the dose rates that all individuals can withstand and the DCRLs that protect populations, there are some individual plants experiencing adverse effects.

## Author Contributions

NC conceived the approach, produced some of the figures, and wrote the first drafts. NW wrote the final draft and contributed some of the figures.

## Conflict of Interest Statement

The Environment Agency (England) and Radioactive Waste Management Ltd. have part funded the research and are potential beneficiaries of the results. The authors declare that the research was conducted in the absence of any commercial or financial relationships that could be construed as a potential conflict of interest.

## Supplementary Material

DATA SHEET S1 Estimated ionizing radiation dose rate through geological time at the Earth’s surface.

DATA SHEET S2 Radical induction-potential from water by different radiation sources through Earth’s history.

DATA SHEET S3 The activity of radionuclides in the environment from the accident at Chernobyl.

DATA SHEET S4 The doses and dose rates used in studies of the effects of IR on plants.

DATA SHEET S5 Oxidizing radicals from O_3_ exposure compared to those from background IR, Chernobyl and UV.
